# Symbolic Number Comparison Is Not Processed by the Analog Number System: Different Symbolic and Non-symbolic Numerical Distance and Size Effects

**DOI:** 10.3389/fpsyg.2018.00124

**Published:** 2018-02-09

**Authors:** Attila Krajcsi, Gábor Lengyel, Petia Kojouharova

**Affiliations:** ^1^Cognitive Psychology Department, Institute of Psychology, Eötvös Loránd University, Budapest, Hungary; ^2^Department of Cognitive Science, Central European University, Budapest, Hungary; ^3^Doctoral School of Psychology, Eötvös Loránd University, Budapest, Hungary; ^4^Research Centre for Natural Sciences, Institute of Cognitive Neuroscience and Psychology, Hungarian Academy of Sciences, Budapest, Hungary

**Keywords:** Analog Number System, number comparison, Weber's law, diffusion model, symbolic numbers

## Abstract

**HIGHLIGHTS**

- We test whether symbolic number comparison is handled by an analog noisy system.
- Analog system model has systematic biases in describing symbolic number comparison.
- This suggests that symbolic and non-symbolic numbers are processed by different systems.

Dominant numerical cognition models suppose that both symbolic and non-symbolic numbers are processed by the Analog Number System (ANS) working according to Weber's law. It was proposed that in a number comparison task the numerical distance and size effects reflect a ratio-based performance which is the sign of the ANS activation. However, increasing number of findings and alternative models propose that symbolic and non-symbolic numbers might be processed by different representations. Importantly, alternative explanations may offer similar predictions to the ANS prediction, therefore, former evidence usually utilizing only the goodness of fit of the ANS prediction is not sufficient to support the ANS account. To test the ANS model more rigorously, a more extensive test is offered here. Several properties of the ANS predictions for the error rates, reaction times, and diffusion model drift rates were systematically analyzed in both non-symbolic dot comparison and symbolic Indo-Arabic comparison tasks. It was consistently found that while the ANS model's prediction is relatively good for the non-symbolic dot comparison, its prediction is poorer and systematically biased for the symbolic Indo-Arabic comparison. We conclude that only non-symbolic comparison is supported by the ANS, and symbolic number comparisons are processed by other representation.

## Representation behind symbolic number processing

### Analog number system

In their seminal work Moyer and Landauer (1967) described that in an Indo-Arabic single digit number comparison task the performance is worse (i.e., reaction time is slower and error rate is higher) when the difference between the two numbers is relatively small (numerical distance effect) or when the numbers are relatively large (numerical size effect). They proposed that the effects are the expression of a general ratio-based effect in which number pairs with smaller ratio are harder to process. This ratio-based performance was thought to be the result of a simple representation working according to Weber's law, termed the Analog Number System (ANS), similar to the representations working behind simple physical feature comparison tasks. Since then, the ratio-based performance (usually measured only with the distance effect) is thought to be the signal of a noisy analog representation working in the background.

The ratio-based performance was also specified with quantitative descriptions. Originally, Moyer and Landauer (1967) demonstrated that the reaction time pattern can be described appropriately with a function used at that time in physical property comparison tasks: a *K* × *log* (*large_number*/(*large_number–small_number*)) function correlates well with the measured reaction time, *r* = 0.75. Later, more precise mathematical descriptions were offered (see Dehaene, 2007 for an extensive mathematical description of the model). According to one of the implementations of these descriptions, the numbers are stored as noisy representation following a Gaussian distribution, and the noise is proportional to the value of the number. This increasing noise can produce the ratio-based performance. For example, the overlap between the representations of two numbers predicts the error rate in a comparison task, or more generally, this overlap predicts the difficulty of the task, expressed as drift rate in the diffusion model (see more details in the Methods section). (This proportionally increasing noise can also be implemented in a logarithmic representation with constant noise on a logarithmic scale).

The ANS is supposed to work behind any number comparison, independent of the notation of the numbers (Dehaene, 1992; Nieder, 2005; Piazza, 2010), because the same ratio-based performance can be observed behind symbolic and non-symbolic tasks (Moyer and Landauer, 1967; Dehaene, 2007), and because overlapping brain areas are activated in symbolic and non-symbolic number processing (Eger et al., 2003; Nieder, 2005). Although there could be differences between the symbolic and non-symbolic number processing, and even there could be two different representations working with different sensitivity (i.e., Weber fraction), both of these stimuli are processed by the same *type* of representations, which representations work according to Weber's law, producing a ratio-based performance (Dehaene, 2007; Piazza, 2010).

The common mechanism and the strong relation between symbolic and non-symbolic processing is also reflected by several findings showing that, for example, the sensitivity of the ANS measured in a non-symbolic dot comparison task correlates with symbolic math achievement (Halberda et al., 2008), or training non-symbolic number processing improves the symbolic number processing (Park and Brannon, 2013). To summarize, it is widely supposed that number processing is supported by a noisy, analog representation, working according to Weber's law, and therefore producing a ratio-based performance in comparison tasks. Also, this type of mechanism works behind both symbolic and non-symbolic number processing, as reflected by many similarities and relations between symbolic and non-symbolic numerical tasks.

### Different symbolic and non-symbolic number processing

However, there are increasing number of findings in the literature suggesting that the symbolic and non-symbolic number processing is not backed by the same representation or by the same type of representations. For example, it has been shown that performance of the symbolic and non-symbolic number comparison tasks do not correlate in children (Holloway and Ansari, 2009; Sasanguie et al., 2014). As another example, while former studies found that common brain areas are activated by both symbolic and non-symbolic stimuli (Eger et al., 2003; Piazza et al., 2004), later works with more sensitive methods found only notation-specific activations (Damarla and Just, 2013; Bulthé et al., 2014, 2015). In another fMRI study, the size of the symbolic and non-symbolic number activations did not correlate, and more importantly, the activation for the symbolic number processing seemed to be discrete and not analog (Lyons et al., 2015a). According to an extensive meta-analysis, while it was repeatedly found that the simple number comparison task (the supposed index for the sensitivity of the ANS) correlates with mathematical achievement, it seems that non-symbolic comparison correlates much less with math achievement, than symbolic comparison (Schneider et al., 2017). In another example, Noël and Rousselle (2011) found that while older than 9- or 10-year-old children with developmental dyscalculia (DD) perform worse in both symbolic and non-symbolic tasks than the typically developing children, younger children with DD perform worse than control children only in the symbolic tasks, but not in the non-symbolic tasks, meaning that the deficit is more strongly related to the symbolic number processing, and the impaired non-symbolic performance is only the consequence of the symbolic processing problems. See a more extensive review of similar findings in Leibovich and Ansari (2016). All of these findings are in line with the present proposal, suggesting that symbolic and non-symbolic numbers are processed by different systems.

Additionally, there are a few alternative models that are in line with these later findings showing that symbolic and non-symbolic number processing is not backed by the same representation or by the same type of systems. In a connectionist model of symbolic number processing, the model successfully explains many phenomena the ANS model cannot handle (Verguts et al., 2005; Verguts and Van Opstal, 2014). Although this model is interpreted as a version of the ANS (Verguts and Fias, 2004; Dehaene, 2007), critically, it does not show the defining feature of the ANS: the model does not produce inherently the ratio-based performance, instead, introduction of the uneven frequency of the digits is necessary to produce the size effect (Verguts and Fias, 2004; Verguts et al., 2005). Thus, the model proposes different type of mechanisms for symbolic and non-symbolic number processing. Another model assumes that primitives (simple representational units) are stored in the long term memory only for the digits (numbers between 0 and 9) (Pinhas and Tzelgov, 2012), but not for other values (Kallai and Tzelgov, 2009; Tzelgov et al., 2009), suggesting a symbolic-only representation. In a third model it was proposed that symbolic numbers can be stored in a Discrete Semantic System (DSS), similar to the mental lexicon or a semantic network. In this system numbers are represented by nodes, and the connections of the nodes reflect the semantic relations of the nodes mostly directed by the numerical distance of the number pairs (Krajcsi et al., 2016). The distance effect might be originated in the semantic relation of the nodes, as was seen in the similar semantic distance effect in a picture naming task (Vigliocco et al., 2002). The numerical size effect could be rooted in the fact that smaller numbers are more frequent than larger numbers (Dehaene and Mehler, 1992), and more frequent numbers can be processed more easily. The DSS model can be easily extended to account for symbolic numerical interference effects as well (Proctor and Cho, 2006; Leth-Steensen et al., 2011; Patro et al., 2014). Thus, the DSS can account for symbolic numerical effects, independent of the non-symbolic number processing.

**Figure 1:**
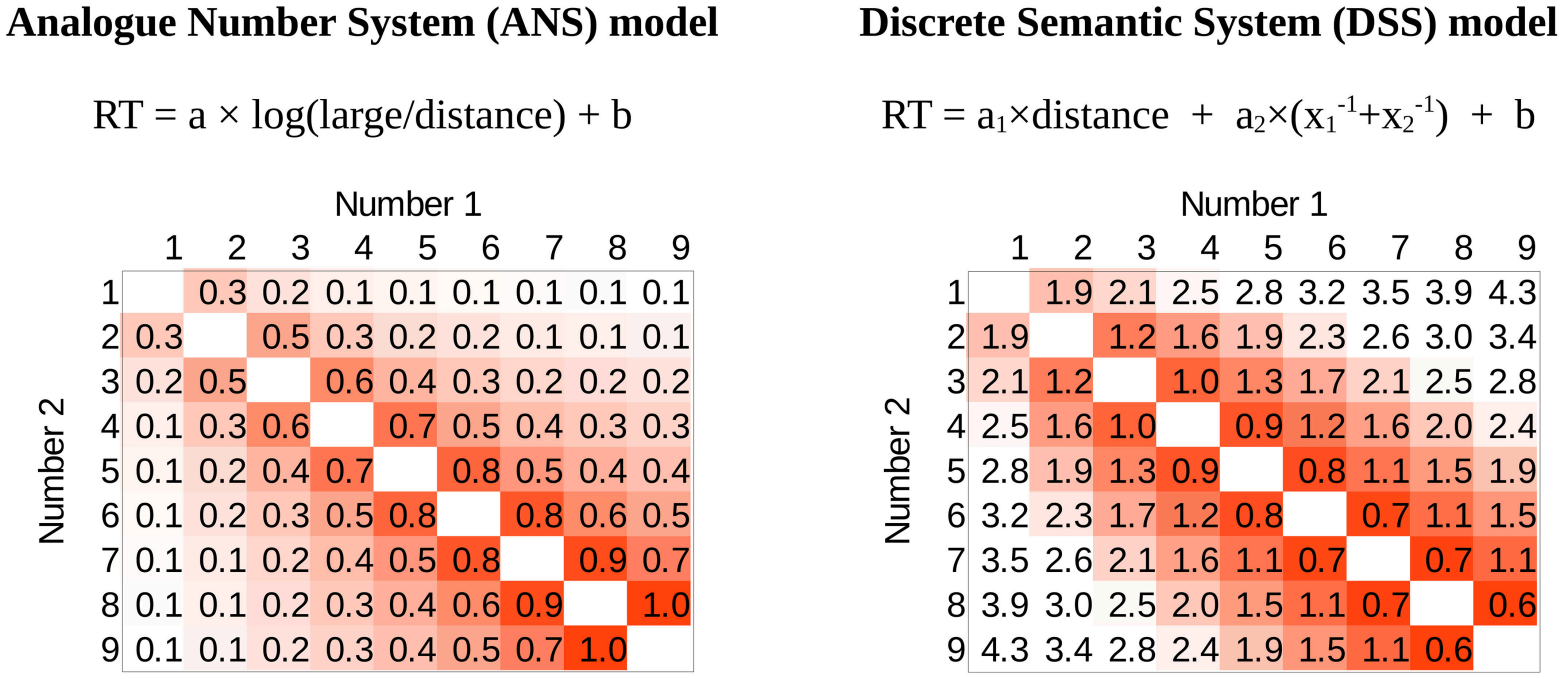
Two possible predictions for the reaction time pattern by the ANS model (based on Crossman, 1955; Moyer and Landauer, 1967) and the DSS model (based on Krajcsi et al., 2016) in a comparison task. The prediction of the models on a full stimulus space in a number comparison task of numbers between 1 and 9. Number 1 and 2 are the two values to be compared. White denotes fast responses, red denotes slow responses (note that numerically the ANS function increases, and the DSS function decreases toward the high ratio, but the direction is irrelevant in the linear fit below). The distance effect can be seen as the gradual change when getting farther from the top-left bottom-right diagonal, and the size effect is seen as the gradual change from top-left to bottom-right. Notations: large: larger number; distance: distance between the two numbers; x_1_ and x_2_: the two numbers; a, a_1_, a_2_ and b are free parameters. In the figures the parameters a and a_2_ are set to 1, a_1_ is 0.4, and parameter *b* is set to 0. See also the Methods section for the interpretation of these heat map graphs.

Importantly, in the DSS account a performance pattern similar to the ANS model can be offered. For example, it is possible that the reaction time could be proportional to the sum of the linear distance effect and the size effect originated in the frequency of the values, which in turn is related to the power of those values (see the justification for this function and similar possibilities in Krajcsi et al., 2016). Figure 1 shows two possible implementations of the ANS and the DSS models, and it reveals that the DSS model might generate a very similar pattern to the one supposed by the ANS model (the correlation of the two presented performance predictions is −0.89).

The similarity of the ANS and the DSS model predictions means that the DSS model could be potentially an appropriate alternative explanation for the observed distance and size effects. Even more importantly, this means that former works investigating whether the ANS model is correct might have found high correlation between the ANS model and the observed performance either because the ANS model is correct, or because it is the DSS model that is correct, and as the ANS model prediction correlates highly with the DSS model prediction, the correlation between the ANS prediction and the performance was only illusory.

To summarize, an increasing body of evidence indicates that symbolic and non-symbolic numbers might be processed by different types of representations, and there could be appropriate alternative models to explain symbolic number processing, which may also question the suitability of former tests.

### The aim of the study

The aim of the present study is to test the appropriateness of the ANS model in comparison tasks more extensively. The appropriateness of the ANS model for both symbolic and non-symbolic notations have been investigated several times, finding that the prediction of the ANS model is similar to the observed performance (Moyer and Landauer, 1967; e.g., Dehaene, 2007). Former studies usually investigated the goodness of fit of the ANS model for the observed performance. However, these former tests are insufficient, because similarity between the ANS model prediction and the observed performance may be caused by alternative models with similar predictions, such as the DSS model. For example, it is possible that in the Moyer and Landauer (1967) study, the *r* = 0.75 correlation between the observed reaction time and the ANS model prediction is the result of a stronger than *r* = 0.75 correlation between the DSS model prediction and the observed performance, and the strong correlation between the DSS model and the ANS model predictions (e.g., *r* = −0.89). Therefore, it is not enough to show that the ANS model's prediction is similar to the observed data, but a more extensive test is needed.

Here we test the appropriateness of the ANS model by investigating whether the ANS model can explain both symbolic and non-symbolic comparison tasks equally well, or whether there are critical differences between symbolic and non-symbolic comparison tasks.^1^ If the ANS account is correct, then one should expect that the ANS model can describe both symbolic and non-symbolic equally well, as suggested repeatedly in the literature (Moyer and Landauer, 1967; Eger et al., 2003; Nieder, 2005; Dehaene, 2007). However, if there are differences between the symbolic and non-symbolic notations, one might suppose that the ANS can describe the non-symbolic comparison appropriately, in line with the fact that non-symbolic stimuli are visual-perceptual as other physical properties processed by other representations working according to Weber's law (Moyer and Landauer, 1967; Dakin et al., 2011; Gebuis and Reynvoet, 2012; Stoianov and Zorzi, 2012), while the ANS model cannot account for the symbolic comparison, as suggested by the alternative symbolic number processing models.

One might question whether this type of test is meaningful, because symbolic and non-symbolic comparison do not necessarily work in the same way, even if the ANS model is correct. For example, there could be additional notation-specific mechanisms that could change behavioral performance, therefore, one cannot expect that the two notations should show the same performance pattern. However, if someone believes that there could be additional components that might influence the behavioral performance, then one must also question whether the findings suggesting ratio-based performance in any comparisons are valid: even if ratio-based performance is observed, the contribution of the hypothesized additional components should be removed, and if that additional component is unspecified, then nothing could be known about the real mechanism in the background. According to this view, the findings of Moyer and Landauer (1967) or any similar results cannot lead to the conclusion that a ratio-based mechanism is working in the background. Overall, one can believe that the current test is invalid, but at the same time it should also be supposed that all tests demonstrating a ratio-based comparison performance are invalid. Even if this viewpoint might seem unusual, it still could be valid. In this case, another types of tests should be found (see for alternative approaches for these tests in Krajcsi et al., 2016; Krajcsi, 2017). But if one thinks that the works that have proposed that ratio-based performance were valid, the present test should be considered to be valid, too.

In the present work we systematically examine whether ANS predicts both symbolic and non-symbolic number comparison performance equally well. Specifically, we examine (1) whether the error rates can be described equally well by the functions derived from the ANS model, (2) whether the reaction time pattern of the two notations fit each other linearly, and (3) whether the diffusion model drift rates of the two notations can be described by the same analog representation. According to the widely accepted version of the ANS model, the model should predict any comparison equally well, because the same ANS-type mechanism processes any numbers independent of their notations. On the other hand, the alternative views might suggest that the ANS should work relatively well only for the non-symbolic notation, but it should work relatively poorly for symbolic notation, because symbolic precise numbers are processed by other mechanisms. Finally, from a methodological point of view, it is also possible that the difference between the ANS and the alternative models is much smaller than the typical noise in the measured data, thus, even if there are differences between the symbolic and non-symbolic comparisons, the signal-to-noise ratio is not high enough to reveal the difference. For this reason only different behavioral patterns of symbolic and non-symbolic comparisons can be conclusive, supporting the alternative accounts, while lack of difference between the symbolic and non-symbolic comparisons could be either due to the correct ANS description or due to the lack of statistical power.

## Methods

Participants compared Indo-Arabic numbers in one condition, and they compared dot arrays in another condition. In both conditions error rate and reaction time were measured.

### Stimuli and procedure

In a trial two numbers were visible on the left and on the right sides of the screen, and participants had to choose the larger one by pressing one of the two response keys. The stimuli were visible until key press. The response was followed by an empty screen for 500 ms, then the next trial started.

In the Indo-Arabic condition the numbers were between 1 and 9, to avoid multi-digit numbers (see footnote 1 for more details). All possible pairings of those values were presented, except ties, resulting in 72 possible pairs. All pairs were presented 10 times, resulting 720 trials in the condition. The order of the trials was randomized.

In the dots condition it is not appropriate to use the same 1–9 range as in the Indo-Arabic condition, because sets with less than five objects can be enumerated fast, which fast enumeration is termed subitizing (Kaufman et al., 1949). Subitizing is not an ANS directed process (Revkin et al., 2008), but it is most probably based on pattern detection (Mandler and Shebo, 1982; Krajcsi et al., 2013). Therefore, to measure the ANS based dot estimation, the 1–4 range should be avoided. One option could be to use only the numbers between 5 and 9, however, this solution would considerably decrease the stimulus space. Instead, another solution was applied: it was not the 1–9 range itself that was kept in the dot condition, but the ratios of the 1–9 range. Because according to the ANS model, it is the ratio of the numbers that determines performance, changing the values should not change the performance if the ratios of the values are kept. Therefore, to avoid the 1–4 range, and to keep the critical ratio-based feature at the same time, all numbers between 1 and 9 were multiplied by 5, resulting in a number range between 5 and 45.^2^ In an array of dots, black and white dots in random positions were shown against a gray background (Dakin et al., 2011), thus, the luminance of the stimuli was not informative about the numerosity. Dots of an array were drawn randomly in a 2 × 2° area, with a dot diameter of 0.2°, therefore, density and convex hull correlated with the numerosity. Although our stimuli do not control all perceptual features that might influence the perceived numerosity, in the current test, non-numerical influence of the decision process is less relevant, because the ANS model suggests that number comparison is handled by an analog system that could be used in any continuous physical feature comparison (Moyer and Landauer, 1967; Dehaene, 2007), hence, in a general sense, any continuous physical feature comparison working according to the Weber's law could be an appropriate task in our test. Additionally, a mixture of visual ratio-based performance and numerosity ratio-based performance should also produce an approximately ratio-based performance, as reflected in the similar psychometric functions of visual comparison and numerical comparison tasks. Therefore, the simple and limited visual control of the stimuli is appropriate for the aim of the current test.^3^ As in the Indo-Arabic condition, all possible pairs were presented 10 times, resulting in 720 trials in the condition. The order of the trials was randomized.

The order of the conditions was counterbalanced across participants.

### Participants

Twenty-four university students gave informed consent and participated in the study for partial credit course.^4^ Four participants were excluded, because their error rates were higher than 1.5 standard deviation + mean error rates at least in one of the conditions (6% in the Indo-Arabic condition and 15% in the dots condition). Among the remaining 20 participants there were 4 males, the age range was 19–24 years, with a mean of 21.0 years.

### Analysis methods

#### Figures used in the results section

To explore the results in more detail, instead of showing the distance and size effects in the traditional way, the full stimulus space is displayed. The left of Figure 1 shows how an ANS predicted pattern would look like. Rows and columns denote the two numbers to be compared, and the cells include the performance for a specific number pair. In this figure larger values (on an arbitrary scale) and darker colors denote worse performance.

**Figure 2:**
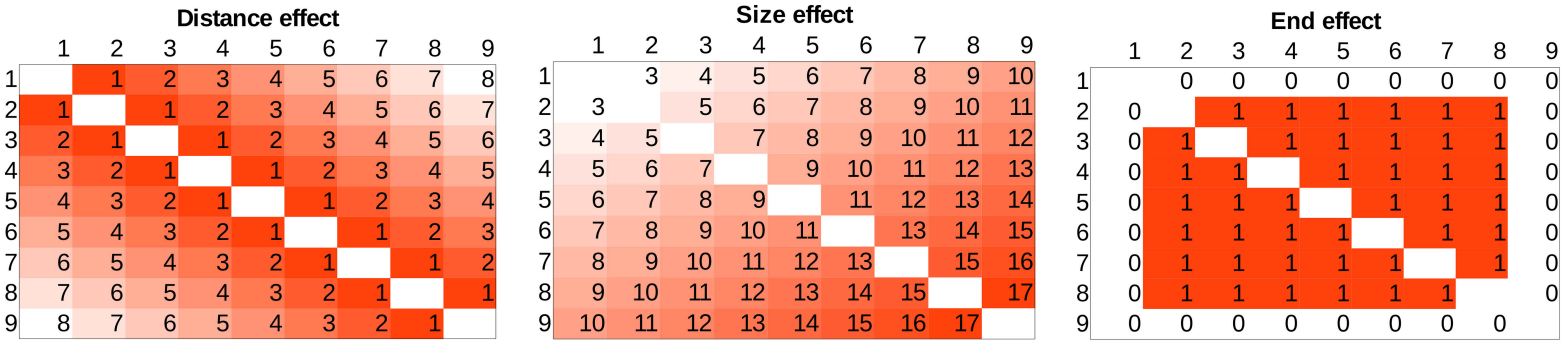
Distance, size, and end effects displayed in the whole stimulus space.

To relate the current figures to the more widely known effects, in Figure 2 some “pure” components of the typical patterns can be seen. Distance effect is displayed as the distance from the top-left and bottom-right diagonal, and size effect is displayed as the distance from the top-left corner along a top-left and bottom-right diagonal. Both effects can also be seen in Figure 1, because the task is harder close to the top-left and bottom-right diagonal (distance effect) and because the task is harder toward the bottom-right corner (size effect). Traditionally, distance and size effects are computed as calculating the mean performance of the cells with the same distance or size values. Sometimes the end effect is also observable (Figure 2, when performance is better with the largest or smallest numbers of the range used in the task (Scholz and Potts, 1974; Balakrishnan and Ashby, 1991; Sathian et al., 1999; Piazza et al., 2002).

These more detailed figures are more appropriate to explore the performance, because (1) any effects that are slightly deviating from the traditional distance and size effects are more visible, and (2) due to the large number of cells systematic patterns can be identified as reliable effects instead of being a random noise, thus, a continuous change in the pattern might signal a specific effect even without statistical hypothesis tests, and random irregularities can be identified as noise.

#### Error rate, reaction time, and drift rate analysis

##### Error rate

In psychophysics, specific functions can be found that describe the error rates in a comparison task based on the stimulus intensities and the Weber ratio (Kingdom and Prins, 2010). These functions are also used in the numerical literature (Dehaene, 2007), serving as a firm base to characterize the ANS model prediction. The functions stem from the model summarized in the Introduction, suggesting that error rate is proportional to the overlap of Gaussian noisy representations. In our analysis we used the function described in Dehaene (2007 Equation 10), which supposes a linear scaling in the ANS,

$$p_{\text{correct}}\left(n_{1,}n_{2}\right)=\int_{0}^{+\infty }\frac{e^{-\frac{1}{2}{\left(\frac{x-\left(r-1\right)}{w\sqrt{1+r^{2}}}\right)}^{2}}}{\sqrt{2\pi }\text{w}\sqrt{1+r^{2}}}dx$$

where *n*_1_ and *n*_2_ are the two numbers to be compared, *r* is the ratio of the larger and the smaller number, and w is the Weber ratio. According to the model this function should work with both symbolic and non-symbolic comparison, although the Weber fraction could be different (Dehaene, 2007). In our analysis the error rates predicted by the specified function above were fit to the group mean of the error rates for both symbolic and non-symbolic comparison for the whole stimulus space.

##### Reaction time

Current models are not straightforward about the reaction time prediction, and former descriptions (such as used in Moyer and Landauer, 1967) are incorrect from the viewpoint of the current models. Still, to test whether former pieces of evidence were used correctly to support the ANS model, we analyzed the reaction time data.

In the last decades the diffusion model (see the Drift rate section in the Analysis methods for details) became a successful and an increasingly popular tool to describe the reaction time of simple decision processes, including psychophysics comparison tasks. However, earlier works used some simpler models to describe the comparison tasks (Crossman, 1955; Welford, 1960; Moyer and Landauer, 1967). From the perspective of the diffusion models these early descriptions are incorrect, because, for example, they did not consider the Weber ratio of the processing system. Still, because evidence using these methods was considered to support the ANS model, in this detailed exploration we also investigate whether these historical tools can support the idea that the ANS processes both symbolic and non-symbolic numbers.

In these early models, there was no clear consensus about the exact function that could describe the reaction time pattern. Psychophysics was more interested in error rates close to the threshold, and much less work investigated the reaction time far from the threshold (Crossman, 1955). For example, the seminal work by Moyer and Landauer (1967) used the *K* × *log* (*large_number/distance*) function^5^, referring to the Welford (1960) paper, which in turn relied on Crossman (1955), however no straightforward solution was proposed then.

Although it is not easy to specify the function that was thought to describe correctly the reaction time pattern of comparison tasks, we can avoid this problem. First, as all models agree that dot comparison is handled by the ANS, dot comparison can be considered as the empirical specification of the required function. Second, in the early models, the specific functions could be fitted linearly to the reaction time: the model can be multiplied by a parameter to fit to the time scale of the comparison process, and a parameter can be added to account for the non-decision time. Moyer and Landauer (1967) also used this method implicitly: they reported Pearson product-moment correlation coefficient between the model and the data, which relies on simple linear regression. The linear transformation between the functions and the data means that the measured patterns should be linear transformations of other measured patterns, too. To summarize, according to the analysis methods of early works, the reaction time patterns of different notations are linear transformations of each other. To test this supposition, we fit the dot comparison reaction time pattern to the Indo-Arabic reaction time pattern. Because both dot and Indo-Arabic comparison data include noise, *R*^2^ is not a suitable index to evaluate the similarity of the patterns. However, looking at the residuals can be more informative: if the two patterns readily fit, then only random noise is expected in the residuals. If, on the other hand, the two patterns differ in shape, then the residuals should show a systematic pattern.

It could be possible to have a more appropriate reaction time pattern with applying the diffusion models (see the next part for details), however, to our knowledge there is no clear consensus among others about the functional relationship between the drift rate and the representational overlap, consequently, the reaction time performance could not be specified easily.

Because the reaction time analysis applied here follows the reasoning of the early analysis, the current results cannot be considered as a reliable test of the ANS model, but we examine whether evidence offered formerly really support the common mechanism for symbolic and non-symbolic number processing.

##### Drift rate

In the recent decades, the diffusion model and related models became increasingly popular to describe simple decision processes (Smith and Ratcliff, 2004; Ratcliff and McKoon, 2008). These models can recover background parameters directing both error rates and reaction times more sensitively. In the diffusion model, decision is based on a gradual accumulation of evidence offered by perceptual and other systems. Decision is made when appropriate amount of evidence is accumulated. Reaction time and error rates partly depend on the quality of the information (termed the drift rate) upon which the evidence is built. Larger drift rate usually results in faster and less erroneous responses. Drift rates are more informative than the error rate or reaction time in themselves, because drift rates reveal the sensitivity of the background mechanisms more directly (Wagenmakers et al., 2007). Importantly for our analysis, observed reaction time and error rate parameters can be used to recover the drift rates (Ratcliff and Tuerlinckx, 2002; Wagenmakers et al., 2007). The drift rates recovered from the behavioral data then can be used to investigate whether they are in line with the prediction of the ANS model.

In the ANS model, like in the case of the error rates, difficulty of the comparison of two properties might depend on the overlap of the two Gaussian random variables: larger overlap leads to worse performance (see the detailed mathematical description in Dehaene, 2007). In the diffusion model framework it is supposed that in a comparison task the drift rate depends purely on the overlap of the two random variables (Palmer et al., 2005; Dehaene, 2007)^6^.

To recover the drift rates for all number pairs in the two notations, the EZ diffusion model was applied, which can be used when the number of trials per cells is relatively small (Wagenmakers et al., 2007). Although this method has several limitations compared to more complex methods (Ratcliff and Tuerlinckx, 2002), (a) all other methods have different limitations, (b) according to current models, the constrains applied in the EZ-diffusion model might not influence the recovered drift rates essentially (although many aspects of the diffusion models are not known yet), and (c) in another numerical task analysis it was found that other tested diffusion models reveal the same pattern as the EZ diffusion model analysis (Kamienkowski et al., 2011). For edge correction we used the half trial solution (see the exact details about edge correction in Wagenmakers et al., 2007). The scaling within-trials variability of drift rate was set to 0.1 in line with the tradition of the diffusion analysis literature.

In the analysis we investigated (a) whether the recovered drift rates are proportional to task difficulty and whether drift rates tend to 0 as the task difficulty increases, and (b) whether drift rates depend purely on the supposed representational overlap, as supposed by the ANS model. As in the case of the error rates, according to the ANS model, these properties should be present in both symbolic and non-symbolic comparisons (Dehaene, 2007).

## Results and discussion

### Mean error rates and mean reaction times

**Figure 3:**
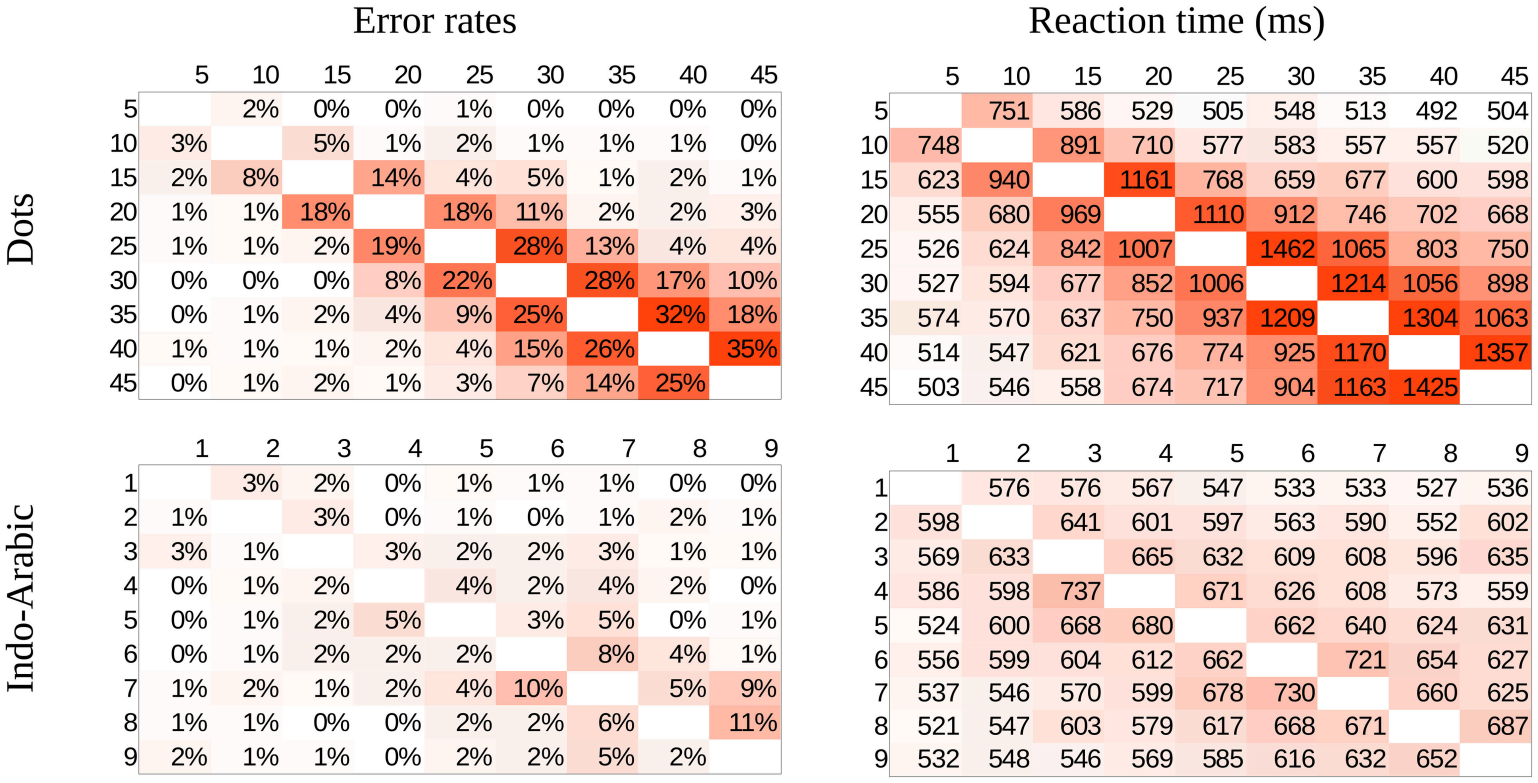
Error rates (left side) and reaction time (right side) in the whole stimulus space in dots (top) and Indo-Arabic (bottom) notations.

Mean error rates and mean reaction times for correct responses were calculated for all number pairs for all participants in the two notations, then mean values across participants were computed (Figure 3). In both notations distance and size effects are visible, the patterns of the two notations seem similar, and based on first visual inspection the patterns could be in line with both the ANS model and the DSS model predictions.

#### Two weber ratios

The error rate results also revealed that the dot comparison is more erroneous than the Indo-Arabic comparison (the mean of the cells are 6.7% for dot notation and 2.0% for Indo-Arabic notation). On one hand, this result is hardly surprising: even common sense would suggest that the exact symbolic comparison is more precise than an imprecise dot array estimation. On the other hand it raises some nontrivial questions. If both types of comparisons are supported by the same representation, how is it possible that the two types of comparisons show radically different error rates and reaction times?

Because the ANS model suggests that the underlying representation works according to Weber's law, a reasonable idea is that the two notations are supported by different Weber ratios: for the Indo-Arabic comparison a more precise, low value is used, while for the dot array comparison a more imprecise, high value is applied. Dehaene (2007) also suggests that the different Weber ratios can be implemented in different neural cells, similar to the simulation in a connectionist model (Verguts and Fias, 2004). In this connectionist model an ANS-like layer represents the values, which layer works according to Weber's law, and after introducing symbolic notation to the network, the nodes of the number layer become more precise. While this explanation about the two Weber ratios seems compelling, there are some problems that are not trivial to solve. (1) Even if the Weber ratio is relatively small, soon it will reach a ratio in which the noise and the error rates will be too high to complete precise comparisons successfully. However, humans can compare numbers with any precision, which would require an unreasonably small Weber ratio. If one argues that there should be a supplementary mechanism that could help with the very small ratio number pairs, then why is its contribution practically invisible as suggested by the ANS model implicitly (i.e., if the Indo-Arabic comparison performance can be predicted precisely by the ANS model, then no other mechanism should have a major contribution to the measured performance)? (2) Actually, as already discussed in the Introduction, the Verguts model cannot be considered as an ANS model, because after introducing the symbolic numbers, the number layer cannot produce the size effect, violating the ratio-based performance which is a defining feature of the ANS model (Verguts and Fias, 2004), and only the addition of number frequency could restore the size effect in the model (Verguts et al., 2005), thus, the model cannot work according to Weber's law after the introduction of symbolic notation. Although none of those problems state that the ANS is incorrect, they indicate that some non-trivial problems should be solved to maintain its coherence.

Although we have not been able to find convincing answers to the questions mentioned so far, in the rest of our analysis we still suppose that the two Weber ratios model is correct, and investigate whether the ANS model with two ratios can explain the Indo-Arabic and dots comparisons equally well. This supposition is in line with the different mean error rate of the two notations, and it reflects the views of the proposers of the ANS model (e.g., Piazza et al., 2004; Dehaene, 2007).

### ANS predictions for the error rates

**Figure 4:**
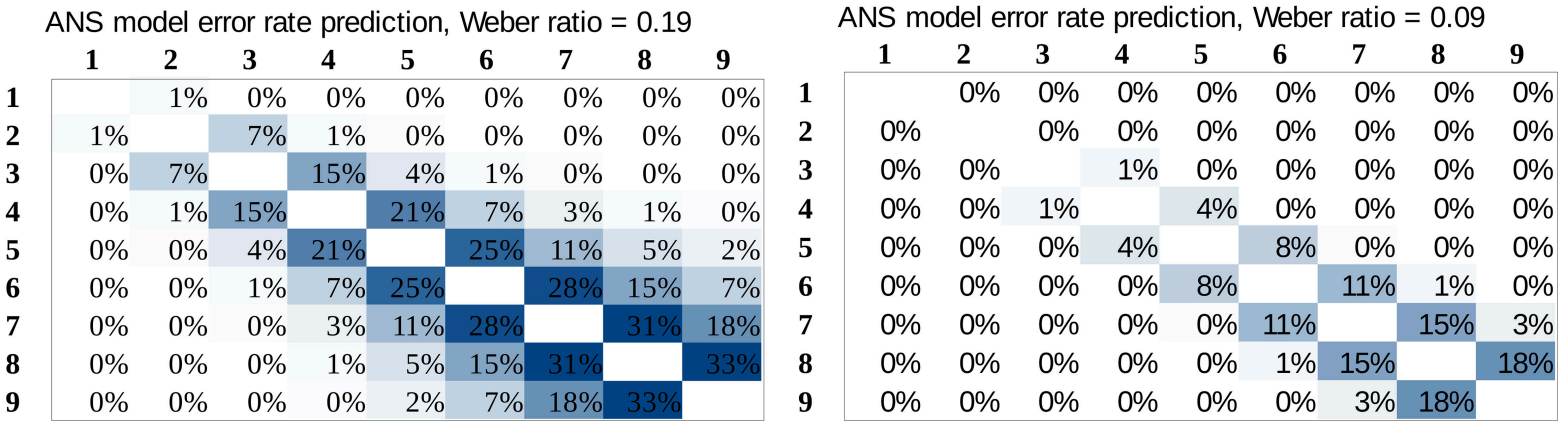
Error rate predictions of the ANS model in our full stimulus space for two Weber ratios. The Weber ratios were determined based on the mean error rates, see Figure 5 and the text.

**Figure 5:**
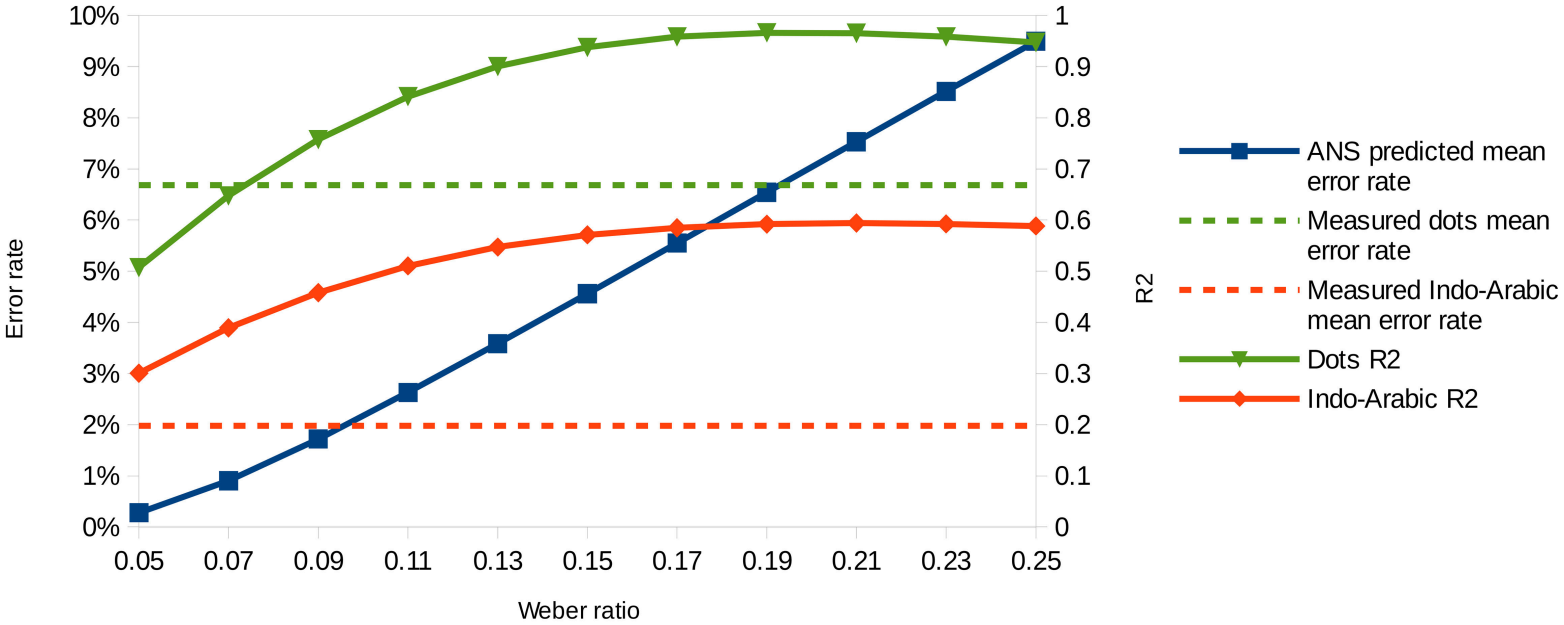
Predicted mean error rates (left y axis) as a function of Weber ratio, and measured mean error rates (left y axis) of the two notations. Goodness of fit (right y axis) as a function of Weber ratio for the dot comparison and the Indo-Arabic comparison.

In the present section we investigate whether the ANS model predicts the error rate patterns in both notations equally well. We calculated the error rate prediction pattern in our stimulus space for several Weber ratios. Two examples can be seen in Figure 4. Weber ratios between 0.05 and 0.25 with a step size of 0.02 were calculated, and fit of the models were calculated for all Weber ratios and for both dot comparison and Indo-Arabic comparison. Figure 5 shows the *R*^2^ values (right y axes) for the dot comparison and the Indo-Arabic comparison as a function of the Weber ratio (x axis).

First, it is important to clarify that the overall *R*^2^ value difference between the two notations is not appropriate to evaluate the ANS model. While the dot comparison reaches its *R*^2^ maximum at around 0.95, the Indo-Arabic comparison *R*^2^ is not higher than 0.6. The different maximum *R*^2^ values can not only be the result of worse overall fit of the ANS model to the Indo-Arabic comparison, but it can also be the result of the smaller error rate in Indo-Arabic comparison. It is reasonable to suppose that the amount of noise is the same in both notations. However, because of the smaller error rate in Indo-Arabic comparison, the number pairs related variability is also smaller. Thus, the Indo-Arabic comparison has a lower signal-to-noise ratio. *R*^2^ shows the percentage of the variance the model can explain of the data, but because of the lower signal-to-noise ratio, the percentage of the variance a perfect model could explain is smaller, thus, the maximum *R*^2^ a perfect model could reach is also lower. Although the *R*^2^ should be lower for a less appropriate model, here the variance of the *R*^2^ is directed more strongly by the signal-to-noise ratio. This is another reason why the overall *R*^2^ cannot be used to contrast the model's prediction in the two notations, but a more indirect analysis is required.

Several properties of the ANS model are important, which properties can be used to assess how correct the model is for the two notations. These properties can also show why a more traditional model comparison method is not sufficient.

**Figure 6:**
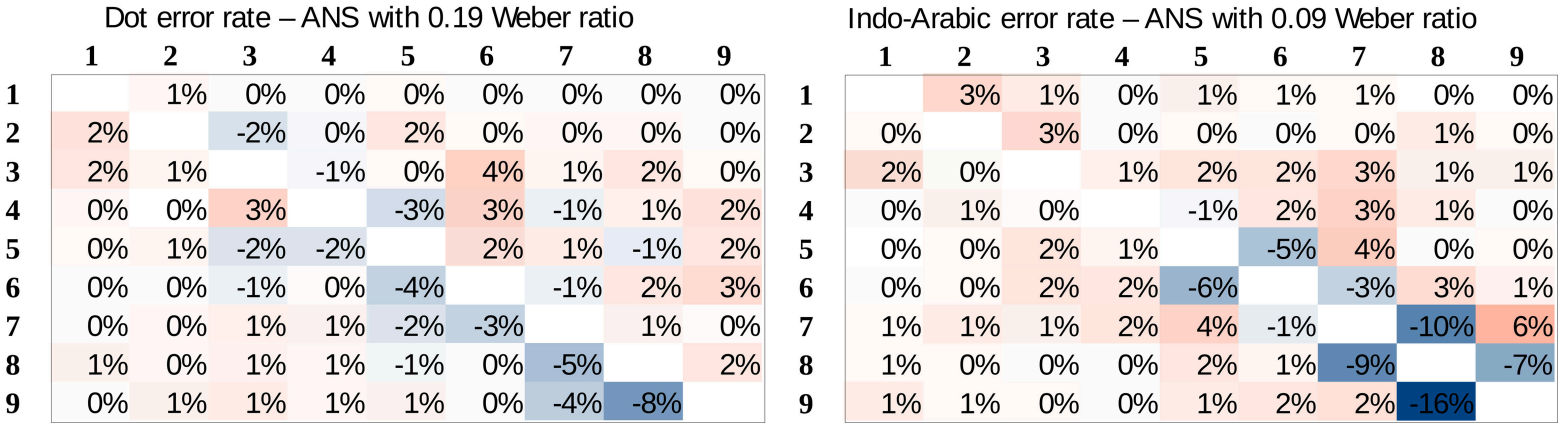
Difference of the measured and predicted error rates for dot comparison (left) and Indo-Arabic comparison (right). Positive values show underestimation of the error rates by the model, negative values show overestimation.

1. **Consistent predicted mean error rates and predicted performance patterns (*R*^2^ values)**. Because the ANS model predicts the mean error rate directly, a model with appropriate Weber fraction should find the mean error rate of the measured performance. Additionally, because according to the ANS model the exact shape of the predicted performance (performance pattern) depends on the Weber-fraction of the representation^7^, it also means that a linear fit of that prediction to the measured data should show the highest goodness of fit, when the model uses the appropriate Weber-fraction. Combining these statements, when the appropriate Weber-fraction is found, (a) the model should show the error rate prediction, and at the same time (b) it should show the highest goodness of fit (e.g., highest *R*^2^ value) reflecting that the model finds the shape of the performance across the stimulus space.
  To determine the Weber ratios for the two notations, we looked for the mean error rates of Weber ratios that are equal with the measured mean error rates of the two notations. Figure 5 shows the predicted mean error rate (left y axis) as a function of Weber ratios (x axis), and the measured Indo-Arabic and dot mean error rates (dashed horizontal lines). Intersections of the prediction (solid line with squares) and the measured data (dashed horizontal lines) specify the Weber ratios of the two notations. According to this, the Weber ratio of the dot comparison should be around 0.19, and the Weber ratio of the Indo-Arabic comparison should be around 0.09. The 0.19 value for non-symbolic stimuli is indeed a typical Weber ratio according to former studies (see for example the results of an extensive measurement in Halberda and Odic, 2014; or the summary of Piazza, 2010 for a review about the development of the Weber ratio). One can note that in the measured data the large ratio cells (e.g., 2 vs. 8, or 10 vs. 45) sometimes show a larger than 0% error rate (Figure 3), which is not in line with the prediction of the model (Figure 4), reflecting a base error rate, which is independent of the specific number pairs. Because the model cannot account for this error rate which is independent of the comparison stage, it could be more appropriate to subtract this base error rate (around 1%) from the measured error rate (lowering the horizontal dashed line on Figure 5). This correction would decrease the Weber ratios by a value around 0.02. All the following results are presented with the 0.19 and 0.09 Weber ratio values, although the same result patterns could be seen with the corrected 0.17 and 0.07 values, too.
  After specifying the Weber ratios of the comparisons for the two notations, one can check if those Weber ratios also show the highest *R*^2^ values. As discussed above, because the goodness of fit should be highest when the Weber ratio is specified correctly (i.e., the model should produce exactly the shape that was measured), the model predicts that the best fit (e.g., the highest *R*^2^) can be obtained with the Weber ratio that is in line with the mean error rate of the notation. With all other Weber ratios the goodness of the fit should be worse. In the dot comparison task the *R*^2^ indeed reaches its maximum around 0.19 Weber ratio, which Weber ratio was predicted based on the measured mean error rate. Thus, the ANS model predicts correctly that the Weber ratio of the best fitting pattern and the Weber ratio based on the mean error rates are approximately the same values. However, in the Indo-Arabic comparison the best *R*^2^ value is around 0.2 Weber ratio, which is much larger than the 0.09 ratio specified with the mean error rate. This suggests that the ANS model cannot predict correctly the shape of the error rate pattern and the mean error rate at the same time in this symbolic comparison.
2. **Predicted error rate patterns**. Based on the specified Weber ratios we can compare the predicted and the measured error rate patterns for the whole stimulus space, which can reveal further details how the ANS model prediction deviates from the measured symbolic comparison data. Figure 4 actually shows the predictions of the model for the Weber ratios with the identified dot and Indo-Arabic Weber ratios, thus, these patterns can be directly compared with the measured data (Figure 3). The difference of the measured and the predicted data can be seen in Figure 6. Because the model predicts directly the error rates, Figure 6 can be considered almost as the residuals after fitting the model to the measured data. Positive values show that the model underestimates the measured error rate, while negative values show that the model overestimates the actual error rate. In both notations the model and the actual data show systematic biases, however, they are qualitatively different in nature. (2a) In the dot comparison the misfit of the model is present because the measured data show an asymmetry related to the order of the stimuli, and the model cannot handle this asymmetry. In small ratio pairs large-small number pairs are responded to with smaller error rates (and faster, see Figure 3) than small-large number pairs. This effect can be the temporal congruity effect, in which large-small order pairs are handled faster than the small-large order pairs when the instruction is to choose the larger value (Schwarz and Stein, 1998). The effect may appear in our data if participants process the left stimulus first, which is consistent with the Western reading direction. The size of the temporal congruity effect is proportional to the difference of the onset of the two values, and disappears when the two stimuli are presented simultaneously (Schwarz and Stein, 1998). This latter property might explain why in our data the effect is only visible when the processing time is slow. It was proposed that the statistical feature of the data could be used to produce the effect: large numbers have higher probability to be the higher number in a pair, and according to this property, the decision criteria may be modified (Schwarz and Stein, 1998). Otherwise the prediction of the ANS model is relatively correct. (2b) On the other hand, residuals in the Indo-Arabic comparison show a completely different misfit. The model supposes that the error rate is very low for most of the number pairs, and error rate increases steeply for small ratio numbers. Instead of this pattern, measured error rates show that the small ratio number pairs do not show such a high error rate, and error rate starts to increase with larger distance in contrast with the model's prediction. These differences can be seen on the residuals as large overestimation for small ratios, and medium underestimation for medium ratios by the model. (These patterns remain if one would use the base error rate corrected 0.17 and 0.07 Weber ratios, although overall the models would underestimate the measured errors.) These observations suggest that while the ANS model predicts the ratio-based comparison error rates relatively correctly (except the order-based preference for the large-small stimuli in low ratio pairs, which asymmetric effect could be an additional effect), the model cannot describe appropriately the Indo-Arabic comparison error rate pattern.
3. **Linear regression parameters of the model**. The found parameters of the fitting procedure shed additional light on how the ANS model fails to explain symbolic comparison data. The ANS error function predicts the error rate directly, therefore, with the appropriate Weber ratio the equation of the fit should be *measured_error* = *1* × *predicted_error* + *0*. How do the parameters change across different Weber values? In the dot comparison task, for example for an incorrectly small 0.07 Weber ratio the fitted function is *2.83* × *model* + *0.04*. This high slope is reasonable, because the small Weber ratio predicts too small error rates that should be increased to fit the measured data. For larger Weber ratio the slope gradually decreases, and with the 0.19 Weber ratio (that was specified with the mean error rate) the function is *0.91* × *model* + *0.01*, in which the slope is rather close to the expected 1 value that the ANS predicts. In the Indo-Arabic comparison for a 0.07 Weber ratio the estimated function is *0.56* × *model* + *0.01*, which is decreasing further as the Weber increases, and for 0.09 Weber ratio the function is *0.37* × *model* + *0.01*. These much lower than 1 slopes reflect that the model predicts too sudden increase with small ratios (as observed in the direct comparison of the measured data and the model), and the fit is better when the model is flattened. Again, linear fit of the different Weber ratio models shows that while the ANS predicts correctly the dot comparison error rates, the model cannot predict the Indo-Arabic comparison.

To summarize, in a more extensive analysis, we found that on one hand the ANS model's prediction is coherent in the dot condition: a 0.19 Weber ratio correctly predicts the mean error rate, the relative shape of the error rates and the specific error rates for the number pairs. On the other hand, in the Indo-Arabic comparison the ANS model predicts a too steeply increasing error rate for small ratios, reflected in incoherent fit results. Again, the ANS model proposes that beyond the Weber fraction differences between the two notations, the same error function should hold for both notations (Dehaene, 2007), therefore, the lack of the precise ANS model description of the symbolic comparison is not the consequence of the notations specific processes. Thus, these results contradict the ANS model in its current form that suggests that both symbolic and non-symbolic comparisons are handled by the same type of representations.

### Linear similarity of the reaction time patterns

**Figure 7:**
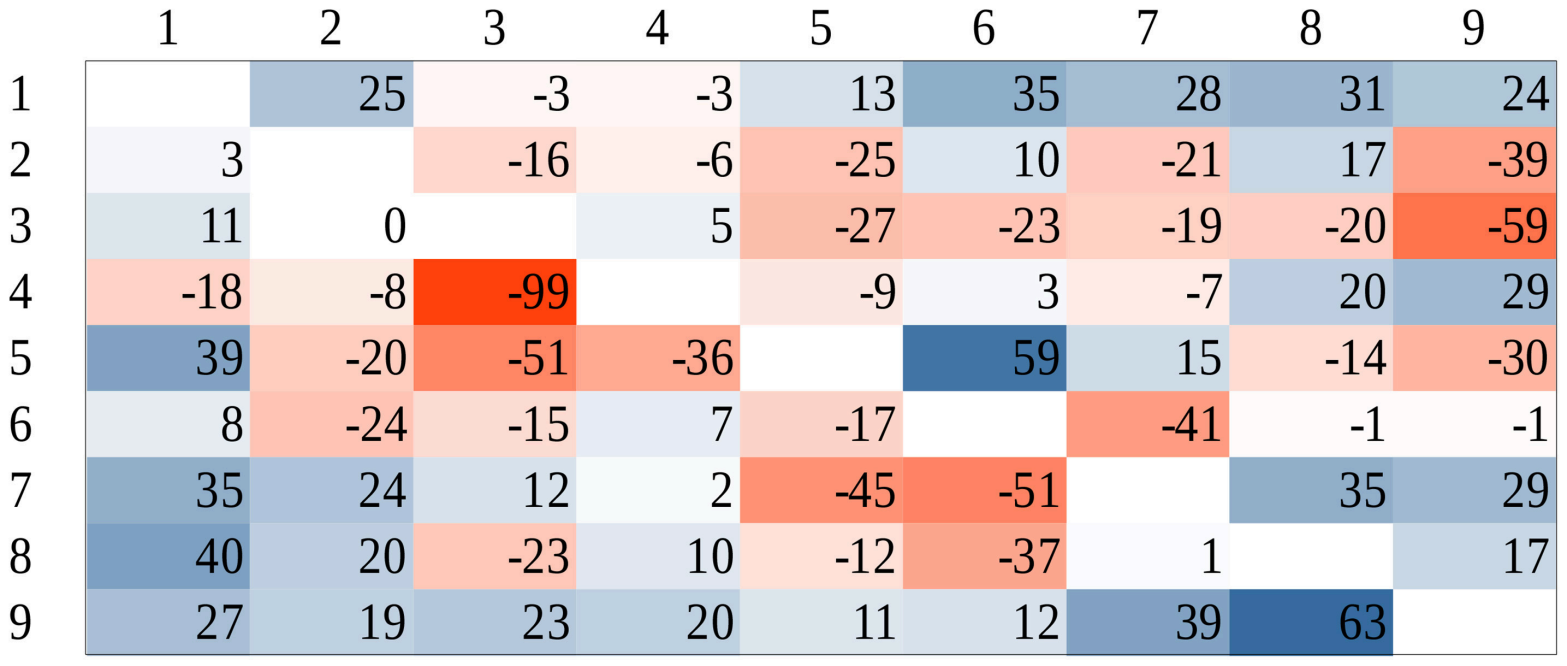
Residuals after fitting dot comparison reaction time to the Indo-Arabic reaction time. Positive values denote higher fitted dot reaction time, negative values denote higher Indo-Arabic reaction time.

Group mean of dot comparison time for the whole stimulus space was fit to the group mean of Indo-Arabic comparison time for the whole stimulus space (right of Figure 3) According to the result, *Indo-Arabic_RT* = 0.17 × *dot_RT* + 474.8, *R*^2^ = 0.684. Residuals of the fit (Figure 7) show an observable systematic pattern. The fitted dot data underestimate Indo-Arabic reaction time for small distance pairs, and overestimates it for large distance pairs. Additionally, the fitted dot data overestimate the cells with 1 and 9 values, similar to an end effect (see Figure 2). To test the presence of these effects in the residuals, multiple linear regression was used with linear distance effect and end effect regressors (see Figure 2), and the residual pattern was used as the dependent variable. Only the end effect regressor was significant (slope is 22.3, *p* = 0.002), while the distance effect was not (slope is 1.3, *p* = 0.452). The statistical lack of the distance effect contradicts the observable pattern, although visual inspection could be unreliable. One source of this contradiction could be the insufficient signal-to-noise ratio, and outliers might decrease the statistical power. After excluding two outlier cells (4-3 and 5-6) the correlation between the linear distance effect and the residuals when both numbers are in the 2–8 range (i.e., without the end effect cells) becomes significant, *r*(38) = 0.28, *p* = 0.015.^8^ Thus, because of the observed systematic patterns in the residuals, the reaction time pattern of the dot and Indo-Arabic comparisons cannot be transformed to the other linearly, contrary to the former descriptions.

Although, as we have discussed, this analysis cannot be considered as a sufficiently precise method, it can be used to judge whether this type of reasoning has been cited correctly to support the common mechanism behind symbolic and non-symbolic number processing. Our results suggest again that this test cannot confirm that non-symbolic and symbolic numbers are processed by the same system.

### Diffusion model analysis

**Figure 8:**
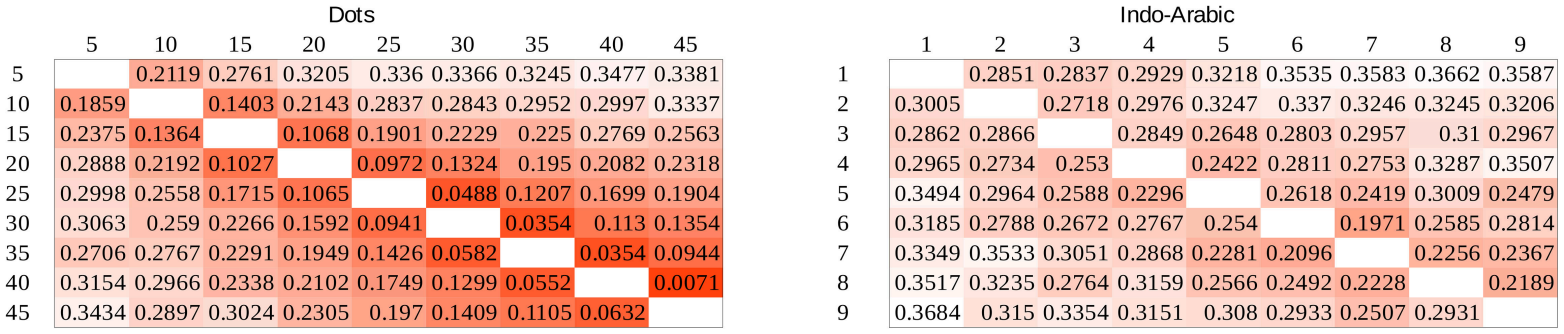
Drift rates in the full stimulus space in dot comparison (left) and in Indo-Arabic comparison (right). Smaller values mean more difficult task.

The diffusion model analysis can be more sensitive than the error rate analysis, and more appropriate than the reaction time analysis by present-day standards. Drift rates for all number pairs and participants were calculated in both notations. The mean drift rates of the participants for the full stimulus space in the two notations are displayed in Figure 8. At first sight it is observable that drift rates show the distance and the size effects in both notations, and the dot comparison is harder than the Indo-Arabic comparison (dot drift rates are smaller), in line with the error rate and the reaction time data.

#### Drift rate and task difficulty

**Figure 9:**
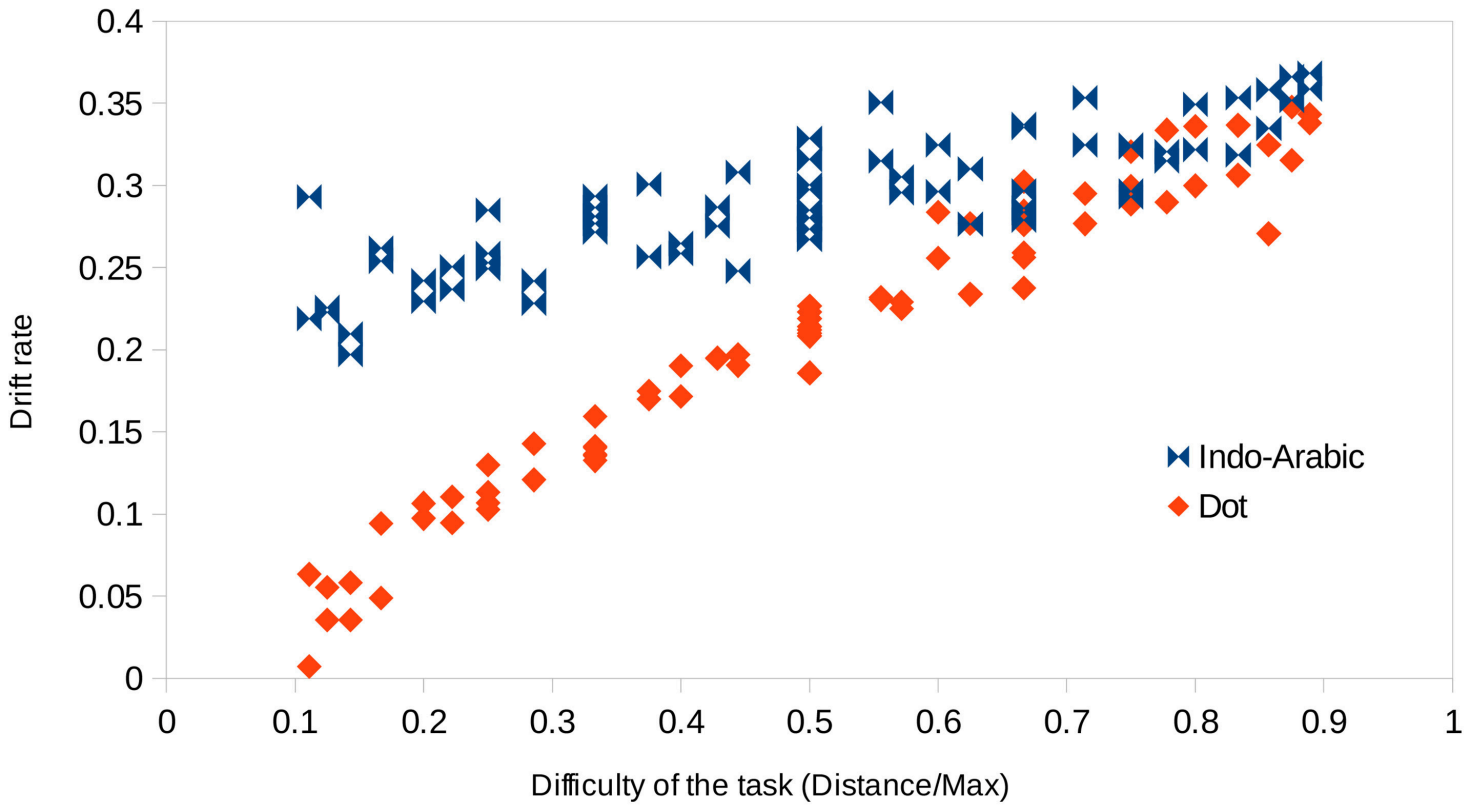
Drift rates of the number pairs as a function of the task difficulty in the two notations.

The values shown in Figure 8 are displayed in a different way in Figure 9. In Figure 9 drift rates are displayed as the function of the difficulty of the task for the two notations. According to the current theories, the observable function in Figure 9 could be proportional, *drift_rate* = *k* × *task_difficulty* (Palmer et al., 2005; Dehaene, 2007), or it could also include a power term as a generalization, *drift_rate* = *k* × *task_difficulty*^β^, although the exponent is often close to 1, thus the first, proportional model approximates the second, power model. In the ANS model, task difficulty is measured as stimulus strength, which is calculated with the *distance/large_number* function as suggested by Palmer et al. (2005) for psychophysics comparison.^9^ There are different properties that should be seen on this figure for any tasks or for tasks solved by an analog system. (1) Easier tasks should show higher drift rates, i.e., in Figure 9 larger values on the x axis should go with larger values on the y axis, showing a positive slope for the curves. This is the case in both notations. However, while in the dot comparison the task difficulty and the drift rate are related more strictly (showing relatively small variance or error around a presumed regression curve), the same relation in the Indo-Arabic notation is much more noisy. (This is not caused by the cells involved in the end effect in Indo-Arabic comparison: after removing those cells, the difference is still visible.) This result is in line with a former study, finding that reaction time is better explained by the ratio in dot comparison task than in Indo-Arabic comparison task (Lyons et al., 2015b p. 1027). This might reflect that while the *distance/large_number* expression suggested by the ANS model might describe the difficulty of the dot comparison relatively well, it might not be applied readily for the Indo-Arabic notation. (2) In an analog representation when the two signals almost completely overlap (i.e., two almost equal properties are shown) the system is hardly able to compare the two properties, which should result in a close to 0 drift rate in the diffusion model (i.e., no evidence is offered for the decision). On Figure 9 the difficulty is measured as *distance/large_number*, and an indistinguishable pair has a *0/large_number* value, which is 0. Thus, when difficulty tends to zero, drift rate should tend to zero, too, therefore, the intercept of the curves should be zero (Palmer et al., 2005; Dehaene, 2007). This is the case in the dot comparison condition, but Indo-Arabic comparison clearly shows a much higher intercept, somewhere around the 0.2 drift rate. This 0.2 intercept is in line with another single digit Indo-Arabic comparison task (Krajcsi et al., 2016), and with the non-zero intercept in multi-digit Indo-Arabic comparison (Dehaene, 2007). Again, these results show that while the dot comparison works according to the ANS model, the Indo-Arabic comparison follows other rules.

The 0 intercept of the dot comparison task also confirms that the use of the EZ diffusion model is at least partly appropriate, because its result correctly reflects an important property of an analog mechanism, therefore validating the EZ method.

Dehaene (2007) analyzed a similar data of an Indo-Arabic multi-digit comparison task, and he also found that the intercept of the drift rate function is larger than zero. We note that a multi-digit symbolic comparison might be a multi-step processing (Hinrichs et al., 1982; Poltrock and Schwartz, 1984; Krajcsi and Szabó, 2012), while diffusion model analysis is appropriate only for short, one cycle processing tasks (Wagenmakers et al., 2007), thus, the diffusion model analysis of multi-digit symbolic numbers should be handled cautiously. Still, independent of this problem, it is important to see how these results, which seemingly contradict the ANS model, could be interpreted to support the classic view. To explain the results in the ANS framework, Dehaene (2007) suggested that there could be two subsystems with two different Weber ratios working in a parallel way, and the interaction of these two subsystems could form the higher than zero intercept and the low slope for the Indo-Arabic number comparison. No further explanation was offered how the two subsystems could form this curve. We think that this two subsystems explanation raises some critical issues. First, it is hard to find why the interaction of two systems will produce high drift rate (and high intercept), when both systems can offer only low drift rates, if the stimuli are almost the same. One reasonable combination of the two drift rates could be the addition of the two values, but adding two small values, that are close to zero (as supposed by the ANS model), cannot result in a relatively high 0.2 value. As a more conceptual phrasing, if none of the two subsystems can differentiate between very small differences, why should any combinations of those analog systems perform much better? Another reasonable combination of the two drift rates is that the higher drift rate should be applied, because the less precise subsystem cannot add any extra information to the already more precise subsystem. Again, it is still not clear how the intercept could increase radically. Another problem with this ANS explanation comes from the low slope of the Indo-Arabic drift rate curve. Dehaene (2007) suggests that in the linear model (*drift_rate* = *k* × *task_difficulty*) *k* is related to the Weber ratio: smaller Weber ratio (higher sensitivity) causes higher slope. Indeed, in the linear model the Weber ratio can be present only in that parameter. Now if we have a *k*_*dot*_ slope observed in the dot comparison task, the *k*_*Indo*−*Arabic*_ slope in the more sensitive Indo-Arabic subsystem should be higher. If those parameters are combined, then again one option is to add the slopes, or another option is to use the larger slope. Both options predict a slope that is larger than the *k*_*dot*_, however, the result shows a smaller value. In a more conceptual rephrase of this problem, the lower slope of the Indo-Arabic comparison suggests a higher (less sensitive) Weber ratio, which contradict the idea that the Indo-Arabic comparison must be more sensitive than the dot comparison. Overall, we cannot see how the ANS model could explain a drift rate curve with high intercept and low slope, and we propose that the analysis of the Indo-Arabic comparison drift rate data as a function of task difficulty is not in line with the ANS or any other representation working according to Weber's law.

#### Drift rate and representational overlap

While in the previous analysis the task difficulty was expressed by the relation of the two numbers, one can also incorporate the Weber ratio. The overlap of the representations of the two numbers can be calculated, that depends on the two values and the Weber ratio. The ANS model has another prediction that can be tested here: according to the model, the representational overlap predicts the drift rates in a comparison task. In contrast with the previous task difficulty vs. drift rate analysis, this relation of the drift rates and representational overlap is independent of the notation, because the different Weber ratios of the two notations are already incorporated in the overlap values.

**Figure 10:**
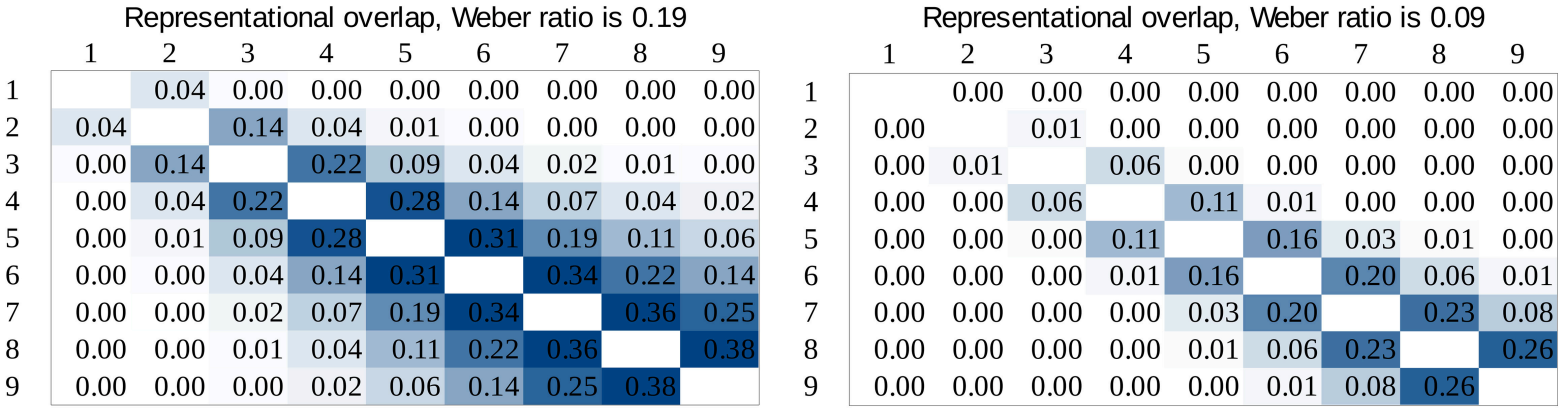
Representational overlap in our stimulus space predicted by the ANS model for Weber ratios 0.19 and 0.9.

To test whether drift rates depend purely on the representational overlap we calculated the representational overlap for all number pairs in our stimulus space for the two Weber ratios specified earlier. To calculate the overlap of two numbers, two Gaussian distributions were created on a linear scale, with the mean of the two numbers to be compared, and standard deviation was the product of the numbers and the Weber ratio (Halberda and Odic, 2014). Representational overlap values can be seen in Figure 10.

**Figure 11:**
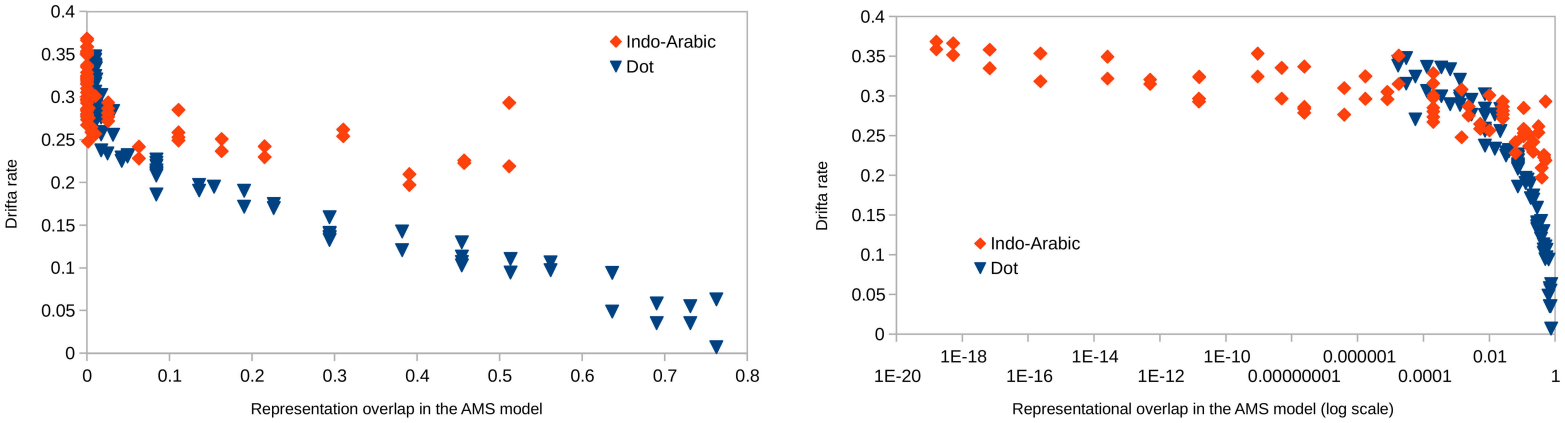
Drift rates as a function of representational overlap in the ANS model in the two notations. Overlap is displayed on linear (left) and logarithmic (right) scale. On the left plot, dot data are shifted by 0.01 to the right not to be covered by the Indo-Arabic data.

Left side of Figure 11 shows the drift rates as a function of representational overlap in the two notations. In the data for small overlaps the signs of the two notations largely overlap, and to show the potentially hidden dot data, dot data are shifted to the right by 0.01. Also, because the data are hard to explore for small overlap values, the same plot is displayed on a log overlap scale on the right of Figure 11. The dot data are not shifted on the latter plot.

According to the ANS model same representational overlap values should result in same drift rate values, independent of the Weber ratio. While for small overlap values the drift rates of the two notations vary in the same range in line with the ANS prediction, for large overlap values Indo-Arabic drift rates are higher than the appropriate dot drift rates, contradicting the ANS model. (This is not caused by the end effect in Indo-Arabic notation: most of the high drift rate values in the large overlap range are not involved in the end effect. Additionally, the same pattern can be seen with the 0.17 and 0.07 Weber rates which are based on the corrected base error rate.) These data, again, show that the ANS model cannot describe the appropriate representations for both notations.

We also note that while there could be uncertainties whether EZ-diffusion model works correctly, in the current analysis all predictions of the ANS model in the dot comparison task proved to be correct, validating the EZ-diffusion model at the same time. This validation confirms that this simple to use diffusion parameter recovery method can be applied appropriately in the current comparison task.

## General discussion

The present work investigated whether symbolic Indo-Arabic number comparison and non-symbolic dot comparison can be described by the same model, as predicted by the widely accepted ANS model, or whether the two notations show systematic differences as suggested by the increasing body of evidence and some alternative accounts of symbolic number processing. Although formerly the ANS description for different notation comparisons has been tested, and the fit was found to be satisfactory, the similarity between the ANS and the recently proposed DSS model predictions required a more rigorous and extensive test.

Our results investigating several properties of the ANS model consistently showed that while the ANS model describe several behavioral aspects of the non-symbolic dot comparison relatively well, the symbolic Indo-Arabic comparison deviated from the ANS description at several points. More specifically, (1) while the ANS model predicts the error rate pattern correctly and consistently for non-symbolic dot comparison, it predicts too high error rates in Indo-Arabic comparison for the small ratio pairs, and too low error rates for medium ratio pairs. (2) The reaction time patterns of the two notations have different shapes which cannot be fitted linearly without systematic residuals, although early description of the comparison task reaction time would suggest a stricter similarity between the two patterns. (3a) In the diffusion model framework, while the dot drift rates are more clearly proportional to the difficulty of the task as defined in the ANS model, the relation between the Indo-Arabic drift rates and the ANS derived task difficulty is noisier. (3b) While the dot drift rates tend to zero when the number pairs become indistinguishable, the Indo-Arabic drift rates remain relatively high, contradicting the supposed functioning of a noisy analog representation. (3c) Across the notations, the drift rates do not show the same values depending on the representational overlap as suggested by the ANS model, showing that the two notation comparisons cannot be described by the same mechanism. All of these results show that (a) non-symbolic dot comparison and symbolic Indo-Arabic comparison do not rely purely on the same type of mechanism, and (b) while the ANS model can describe the non-symbolic dot comparison, it cannot describe the symbolic Indo-Arabic notation.

One might wonder whether alternative forms of the ANS model could give an account for our findings, either by modifying the specific functions utilized in the present analyses or by conceptually modifying the model. At least one aspect of our results questions whether this is possible. In Indo-Arabic number comparison the drift rate does not tend to zero when the stimuli become almost indistinguishable, which result cannot be explained by any analog representation working according to the Weber's law. This is an analogous form of the problem that it is difficult to explain how the imprecise ANS could be responsible for precise number processing. If the EZ diffusion model recovered appropriately the drift rates (we indeed found that many properties of the non-symbolic drift rates are in line with the psychophysics model, which validates the EZ model), then the symbolic number comparison cannot be processed by any analog representation working according to the Weber's law, which is a defining feature of the ANS model. Thus, we argue that the ANS model cannot be modified to account for the present findings.

One might also wonder whether shorter presentation of the dot stimuli could modify the results, because that could ensure that the diffusion model analysis handles a single step decision process instead of a multi-step counting process. However, the relatively precise prediction of the ANS model in dot comparison reflects that the current stimuli are successful enough to show the appropriateness of the ANS model, and further refinements can only improve this appropriateness. More generally, because the current design and stimuli were already appropriate to show that the ANS model describes non-symbolic comparison correctly, there is no need to further improve the current methods using the non-symbolic stimuli.

Beyond the current empirical results, suggesting that only non-symbolic comparison seems to be supported by an analog representation, but not symbolic comparison, we briefly summarize some non-trivial key problems of the ANS model explaining symbolic number processing. (1) As we have mentioned, how could an imprecise system, as the ANS, solve precise symbolic comparison? Even a smaller Weber ratio (more sensitive system) is inappropriate to solve this issue. (2) If a supplementary precise system helps to solve precise symbolic comparison, why is this system invisible in a sense that dominant part of the variance in the comparison performance is purely influenced by the ANS? Additionally, why is the ANS thought to dominantly influence performance in cases when it cannot solve the problem at all? (3) If the supplementary precise system has an effect on the performance, how do we know by looking at the performance that the ANS is also activated in a comparison task? If performance is partly comprised of a hypothetical precise system, then without specifying that precise component, one can not find the rest of the performance that could support the ANS processing either.

To summarize, all of our results show that symbolic and non-symbolic comparisons show several critical differences, and while the ANS model can successfully describe the non-symbolic dot comparison, it cannot account for many features of the symbolic Indo-Arabic comparison. Therefore, we argue that while non-symbolic comparison is supported by the ANS, symbolic comparison and number processing is supported by an alternative system. Further research can confirm whether the increasing amount of data suggest correctly that symbolic and non-symbolic numbers are processed by different types of systems, and if so, what representation is utilized to process symbolic numbers.

## Ethics statement

All studies reported here were carried out in accordance with the recommendations of the Department of Cognitive Psychology ethics committee with written informed consent from all subjects. All subjects gave written informed consent in accordance with the Declaration of Helsinki.

## Author contributions

All authors listed, have made substantial, direct and intellectual contribution to the work, and approved it for publication.

### Conflict of interest statement

The authors declare that the research was conducted in the absence of any commercial or financial relationships that could be construed as a potential conflict of interest.

## Footnotes

^1^A similar investigation of symbolic and non-symbolic comparisons testing against the ANS model was done by Dehaene (2007), however, in that study multi-digit Indo-Arabic numbers were utilized. When comparing multi-digit symbolic numbers one might process the numbers power by power, and holistic ANS processing of the number cannot be guaranteed (Hinrichs et al., 1982; Poltrock and Schwartz, 1984; Krajcsi and Szabó, 2012; Huber et al., 2016), therefore, in such a test multi-digit symbolic numbers should be avoided. The present work utilizes only single digit Indo-Arabic numbers.

^2^One might raise that this way the two notations do not use the same number ranges, consequently, the two conditions are not comparable. However, it is important to highlight that the current work tests the ANS model, and in this specific test any modifications that are in line with the ANS model are appropriate. If the ratio-based transformations were not allowed, it would already mean that the ANS model is incorrect, therefore, no further test would be needed.

^3^Similar to the reasoning in the previous footnote, we take advantage of the fact that this work is an ANS test, and any addition that is in line with the ANS model, is allowed. If one questions that the number-based comparison performance has different properties than physical feature comparison performance, then the ANS model itself is questioned, therefore, no further test would be needed.

^4^Because it is impossible to tell what effects sizes can be expected, or even what properties could differ between the two notations, it is not possible to specify an appropriate sample size in advance. Approximately 25 participants were set as a convenient sample size where the most important effects are firmly observable, but no reasonable prediction could be made regarding the reliability of yet unknown differences between the notations.

^5^In the Moyer and Landauer (1967) paper the *K log* (*large_number*/*large_number -small_number*) function can be found, without the necessary brackets around the large-small term, but most probably the calculation was performed with the correct function.

^6^According to the current models, it is only the drift rate that is relevant in comparison performance (Palmer et al., 2005; Dehaene, 2007). For example, non-decision time is not relevant in the distance effect, because it is not related to the comparison phase (Dehaene, 1996). Similarly, decision threshold is believed to be mainly modulated by the speed-precision instruction (Smith and Ratcliff, 2004) and not by the properties of the stimuli of specific trials. Although it is rare that other than the mean of the performance is investigated in a study, Rouder et al. (2005) measured reaction time properties as parameters of a 3-parameter Weibull distribution. They found that distance effect modified the scale parameter, but not the shape or location parameters. While the relation of the diffusion model generated performance distribution and the Weibull distribution is not fully understood (Rouder et al., 2005), drift rate change of the diffusion model can result in scale parameter change but not in shape or location parameter changes in the 3-parameter Weibull distribution (Rouder et al., 2005), in line with the idea that it is the drift rate that is related to the numerical distance effect.

^7^In other words, ANS predicted performance patterns with different Weber-fraction, e.g., the two error rates shown in Figure 4, cannot be fitted perfectly with a linear transformation.

^8^One might suggest that the apparent distance effect in the residuals could be the artifact of fitting the dot data to the Indo-Arabic data with the end effect in the Indo-Arabic notation, and with the lack of the effect in the dot notation: because there is a stepwise change at the edge of the Indo-Arabic stimulus space, the “outer end” of the fitted distance effect will be lowered, creating a higher slope in the fitted line and a gradually increasing effect in the residuals (a distance like effect). However, such an artifact should underestimate large distance cells, while our data show an overestimation for those cells. Therefore, the distance effect in the residuals cannot be the artifact of the end effect in the Indo-Arabic notation.

^9^Dehaene (2007) suggests that the difficulty of the task could be expressed as the logarithm of the ratios of the numbers, although that description is not entirely explicit how this function was found. One possibility is that this function was the one that could offer a linear relation between the difficulty of the task and the drift rates presented in that description. We also tested our data with the log(ratio) task difficulty scale, and the results could not be described neither with the proportional model (the curve is clearly non-linear), nor with the power model (the model strongly overestimates the drift rates for the easy tasks). However, Dehaene (2007) (a) used a more restricted diffusion model parameter recovery method, than the EZ diffusion model (although in the same paper EZ diffusion model was also used, its detailed results were not reported), and (b) he analyzed multi-digit number comparison. These differences can explain why a different expression was found as the measure of the task difficulty.
